# Cyclooxygenase-2 Overexpression and its Association with Histopathological Features of Human Malignant Melanoma

**DOI:** 10.30699/ijp.2025.2042091.3357

**Published:** 2025-03-10

**Authors:** Nasrin Shayanfar, Zeinab Gholizade, Fatemeh Montazer, Kambiz Kamyab, Aram Nazari, Ali Zare Mirzaie

**Affiliations:** 1 *Department of Pathology, School of Medicine, Iran University of Medical Sciences, Tehran, Iran*; 2 *Department of Pathology, School of Medicine, Tehran University of Medical Sciences, Tehran, Iran*

**Keywords:** Malignant melanoma, Cyclooxygenase-2, Prognostic factors, immunohistochemistry

## Abstract

**Background & Objective:**

Melanoma is one of the most common types of skin cancer with an annually increasing mortality rate. Cyclooxygenase-2 (COX-2) plays an imperative role as a cancer biomarker in the biosynthesis of prostaglandin and thromboxane during inflammatory reactions. Its overexpression has been demonstrated in various cancer types, including melanoma. However, its clinicopathological association with melanoma is controversial. We aimed to immunohistochemically evaluate COX-2 expression in malignant melanoma (MM) tumors.

**Methods:**

In this cross-sectional retrospective study, blocks from patients with MM who were referred to Rasool-Akram and Razi hospitals between 2011 and 2020 were collected and immunohistochemically evaluated using COX-2 antibody. The intensity and percentage of COX-2 expression was determined in tumoral tissues, and its association with clinical and histological features of patients were evaluated.

**Results:**

A total of 39 patients diagnosed with MM were included in this study, of whom 20 (51.3%) were male and 19 (48.7%) were female, with an average age of 57.28 ± 14.37 (range 14-87 years). The most common histological subtypes were acral melanoma (30.8%) and nodular melanoma (20.5%). The most common locations of tumor involvement were the lower (33.3%) and upper limbs (23.1%). Ulcers and vascular-lymphatic invasion were observed in 33.3% and 5.1% of cases, respectively. 38.5% of tumors were in level IV according to Clark’s level. Elastosis was present in 13% of samples. Approximately 87% of MM samples showed COX-2 expression, 61.5% of which were strong. There was a significant association between COX-2 expression and tumor location (P = .04).

**Conclusion:**

**Our findings highlight that the COX-2 protein is considerably expressed in MM tumors. Therefore, therapeutic strategies with the aim of targeting COX-2 might be considered in the prevention or treatment of MM. However, it remains to be explored whether COX-2 might be a prognostic marker of MM.**

## Introduction

Melanoma is one of the most aggressive and dreaded types of cancer, killing approximately 50 000 cases worldwide every year because of its rapid progression and acquired therapy resistance (1). Most diagnosed cases represent the cutaneous form as the most common cause of 65% of all skin malignancy-related deaths (2). Primary melanomas can be presented as extracutaneous, ocular, mucosal, gastrointestinal, genitourinary, leptomeningeal, and lymphatic (1). The association between exposure to ultraviolet (UV) radiation and melanoma development is extremely complex, as intermittent sun exposure greatly increases the risk of melanoma (1). Melanoma is often diagnosed through clinical evaluation of pigmentation by healthcare professionals (1, 2). Marked cellularity, asymmetry, and poor circumscription are considered architectural characteristics of Malignant Melanoma (MM) (1, 2). Melanoma was diagnosed using cytological aspects, including irregular and thick nuclear membranes and prominent nucleoli. Reduction in exposure to UV light has been announced as a preventive procedure for MM. Hence, early diagnosis of this cancer greatly reduces both short- and long-term morbidity and mortality rates. 

Although in clinical practice, tumor stiffness and mitotic rate have been used as reliable prognostic factors in MM, typical therapies include surgery, gene therapy, chemotherapy, and immunotherapy. However, more reliable biomarkers are desired to predict melanoma treatment response. Among several inflammatory mediators, cyclooxygenases (COXs) appear to be implicated in cancer (3). COXs are enzymes that catalyze the first rate-limiting steps that mediate the arachidonic acid from membrane phospholipids into prostaglandins (PG) via the sequential reactions of cyclooxygenation and peroxidation (4-6). 

Three isoforms of COX have been identified: COX-1, which is ubiquitously expressed in most tissues mediating prostaglandin synthesis and homeostasis in normal physiological processes; inducible COX-2, which is undetectable in normal tissues but is upregulated during both inflammation and cancer; and COX-3, which remains to be elucidated but is normally expressed in the brain and spinal cord (3, 7, 8). The human COX-2 gene encodes a protein containing 604-AA, which is located in the 1q25.2-q25.3 locus with a length of 8.3 kb and 10 exons. Domains of epidermal growth factor (EGF) and membrane binding are placed inside the COX-2 protein near the N-terminus head (4). 

The COX-2 isoform is typically triggered by several extra- or intracellular stimuli, including proinflammatory cytokines, growth factors, mitogens, and hormones (5, 9). It is secreted into the tumor environment by cancer-associated fibroblasts, type 2 macrophage cells, and cancer cells, and its overexpression in malignant tumors may be closely associated with tumorigenesis and metastasis (10-12). The relationship between COX-2 upregulation and tumorigenesis, mainly in solid tumors modulated by various mechanisms similar to cancer stem cells (CSCs), causes resistance to apoptosis, increase in PG, cell proliferation, angiogenesis, inflammation, invasion, and metastasis (4, 13, 14). Increased COX-2 expression has been shown to have prognostic potential in various types of human malignancies such as colon (15, 16), breast (17, 18), lung (19, 20), gastric (21, 22), esophagus (23, 24), pancreas (25), bladder (26), prostate (27), ovary (28, 29), cervix (30, 31), gliomas (32, 33), osteosarcomas (34, 35), and Hodgkin's Lymphomas (36). 

Considering the high mortality rate of melanoma, new diagnostic and therapeutic approaches are required. There are a limited number of studies that evaluated the expression of COX-2 in melanoma. The findings indicated an increase in the expression of this marker in MM, and the effect of COX inhibitors has been applied for treating melanoma (4). These findings prompted us to investigate the level of COX-2 expression in MM and the relationship between the expression of this marker and prognostic factors in melanoma, including depth of invasion, tumor thickness, mitosis rate, lymphovascular invasion, lymphocyte infiltration, and tumor staging. In view of the fact that COX-2 also reacts to X-rays, in this study, the association between COX-2 expression and different types of melanomas will also be investigated according to the amount of optical damage (elastosis).

## Materials

### Patients and tissue specimens

In this retrospective cross-sectional study, 39 formalin-fixed paraffin-embedded (FFPE) tissue samples were collected from patients with MM who were referred to two university-based referral hospitals (Rasoul-Akram and Razi) in Tehran, Iran, from 2010 to 2019. Tumor tissue samples of MM were re-evaluated in terms of histopathology. The nonrandom sampling method was applied based on the available samples. The related clinicopathological and demographic factors of patients, including age, gender, tumor location, histologic subtype, ulcer, Clark’s level, Breslow thickness, lymphovascular invasion, tumor lymphocytes, number of mitoses, pT stage, and elastosis, were extracted from patient’s medical records. The Hematoxylin and Eosin (H&E) stained slides of the relevant tissue blocks were re-examined and categorized according to the new WHO 2018 classification. The best tissue areas were selected for staining. In the next step, the paraffin blocks were cut using a microtome. 

Written informed consent was obtained from each patient in advance. According to the ethical and legal standards, sampling was made anonymous, and ethical approval was obtained by the Ethics Committee of the Iran University of Medical Sciences (with registration code IR.IUMS.FMD.REC.1399.818) to use the patient’s tissues while keeping patient’s data confidential. 

### Immunohistochemistry (IHC) technique

The MM tissue sections were stained to evaluate the expression level of COX-2 using IHC, as described previously (37). Briefly, after tissue sectioning, all slides were placed in an oven at 60°C for 10 to 15 minutes to dewax. The steps of deparaffinization, rehydration, and blocking of internal tissue peroxidase in tumor tissues were performed in xylene and serial ethylic alcohol (absolute and 96 degrees) and methanol (containing 3% hydrogen peroxide), respectively. The slides were then rinsed in Tris-buffered saline (TBS) three times. For antigen retrieval, the tissue slides were then placed in an autoclave for 20 minutes in a container containing a suitable buffer (citrate PH=6). Subsequently, the tissues were exposed to the primary antibody (Anti-COX-2, clone SP21, rabbit monoclonal antibody; ready to use) for 4 hours at RT. Two-step polymer HRP anti-mouse/rabbit was applied as a secondary antibody on slides for 10 minutes at room temperature. After a three-step wash in TBS, slides were counterstained with Mayer’s hematoxylin dye for 3 minutes, dehydrated in serial ethylic alcohols, cleared in xylene, and mounted for evaluation. The stained slides were then interpreted under a light microscope by a pathologist. Non-tumoral tissues such as normal epidermis and vascular endothelial cells were used as internal negative and positive controls, respectively. 

### Staining categorization and scoring

The staining of COX-2 on MM sections was individually interpreted and scored by two pathologists (N.SH and Z.GH) using a double-headed light microscope blinded to the clinical and pathological features of the patients, and a consensus between pathologists was imposed. The expression level of COX-2 was evaluated using two scoring systems, namely, the intensity of staining and the percentage of positive tumor cells. The intensity of staining was recorded as follows: 1, negative or non-staining; 2, weakly staining; 3, strong staining. The percentage of positive cells was divided into three groups as follows: 1, less than 10% of the cells; 2, 10% to 50% of the cells; and 3, more than 50% of the cells were stained. The degree of optical damage was evaluated by examining the degree of solar elastosis, such that grade 1 is described by the appearance of single elastic strands, grade 2 by bundles of fibers, and grade 3 by basophilic material that does not have fibrillar tissue. 

Clark’s classification was used to classify the depth of melanoma invasion, which is defined as follows: I, intraepidermal tumor only (eg, melanoma in situ); II, the tumor is inside the papillary dermis, but it does not fill it; III, the tumor fills the papillary dermis; IV, the tumor invades the reticular dermis; and V, the tumor invades the subcutis. The Cap Cancer Protocol 2020 was used to check the variables of this study. The obtained information was then recorded and analyzed by choosing the appropriate statistical method. 

### Statistical analysis

All data were statistically analyzed using SPSS software version 21.0 (SPSS, Inc., IBM Corp, USA). Qualitative data were reported as percentage and frequency, and quantitative data were reported as Mean ± SD. Chi-square test was used to check the association between qualitative variables. The t-test was used to compare two means, and the ANOVA test was used to compare more than two means. If the data distribution was not normal, nonparametric tests were used. Regression models were applied to control confounders. A statistical significance level of 0.05 will be considered in all parts of the analysis.

**Table 1:** Shows histopathological characteristics of malignant melanoma tumors.

Histopathological characteristics		Number (%)
Ulcer	Yes	13 (33.3)
	No	22 (56.4)
	Not assessable	4 (10.3)
Lymphovascular invasion	Yes	2 (5.1)
	No	33 (84.6)
	Not assessable	4 (10.3)
Clark level	I	1 (2.6)
	II	7 (17.9)
	III	9 (23.1)
	IV	15 (38.5)
	V	2 (5.1)
	Not assessable	5 (12.6)
Intra-tumoral lymphocytes	Negative	15 (42.9)
	Low	16 (45.7)
	High	4 (11.4)
T stage	1	6 (18.8)
	2	10 (31.3)
	3	12 (37.5)
	4	4 (12.5)
Elastosis	Negative	34 (87.2)
	Grade I	4 (10.3)
	Grade II	1 (2.6)
Breslow thickness	≤2	18 (56.3)
	>2	43.7)

## Results

### Patients’ clinicopathological and tumor histopathological features

A total of 39 patients diagnosed with MM were included in this cross-sectional study, of whom 20 (51.3%) were male and 19 (48.7%) were female, with an average age of 57.28 ± 14.37 years (range, 14-87 years). The most common locations of tumor involvement were the lower (33.3%) and upper limbs (23.1%), scalp and neck (17.9%), face (12.8%), trunk (10.3%), and external ear (2.6%). In terms of histological subtype, the most common types were acral melanoma (30.8%), nodular melanoma (20.5%), superficial spreading types (15.4%), Lentigo maligna (12.8%), unclassified (7.7%), in situ types (7.7%), and desmoplastic (2.6%) and metastatic types (2.6%). Ulcers and vascular-lymphatic invasion were observed in 33.3% and 5.1% of cases, respectively. 38.5% of tumors were level IV, according to Clark’s level. Elastosis was present in approximately 13% of samples. Infiltrating lymphocytes inside the tumor was present in approximately 57% of the samples, and 37.5% of the tumors were in the pT3 stage. No mitosis was observed in 14 samples (36%), and the highest number of mitoses observed in one sample was 14 per mm^2^. The average Breslow thickness of tumors was 2.85 ± 2.37 mm, with a median of 2 mm and a range of 0.5 to 11 mm.

### COX-2 Expression levels and its association with clinicopathological features of patients with MM

The cytoplasmic expression of COX-2 was evaluated using the IHC method on MM tumor sections in terms of the intensity of staining and percentage of positive tumor cells. Approximately 87% of MM samples showed COX-2 expression, and 61.5% of them had strong expression of this marker. In addition, in 64.1% of samples, COX-2 expression was present in more than 50% of tumor cells. COX-2 staining was detected in MM cases with different intensities. 

The association between COX-2 expression and clinicopathological features of patients with MM was analyzed using Pearson’s χ2 test (Table 2). There was a significant association between the expression percentage of COX-2 in tumor cells and tumor location (P = .046), but its expression in facial tumors was significantly lower than that in tumors in other body areas. In terms of the tumor histological subtypes, although there was no statistically significant difference between the intensity and percentage of COX-2 expression in different groups, its expression level in melanoma in situ and Lentigo maligna was lower than that in other subtypes. In the case of Clark level, there seems to be an increasing trend in the intensity and percentage of staining with an increase in the level of this clinicopathologic factor. 

**Fig. 1:**
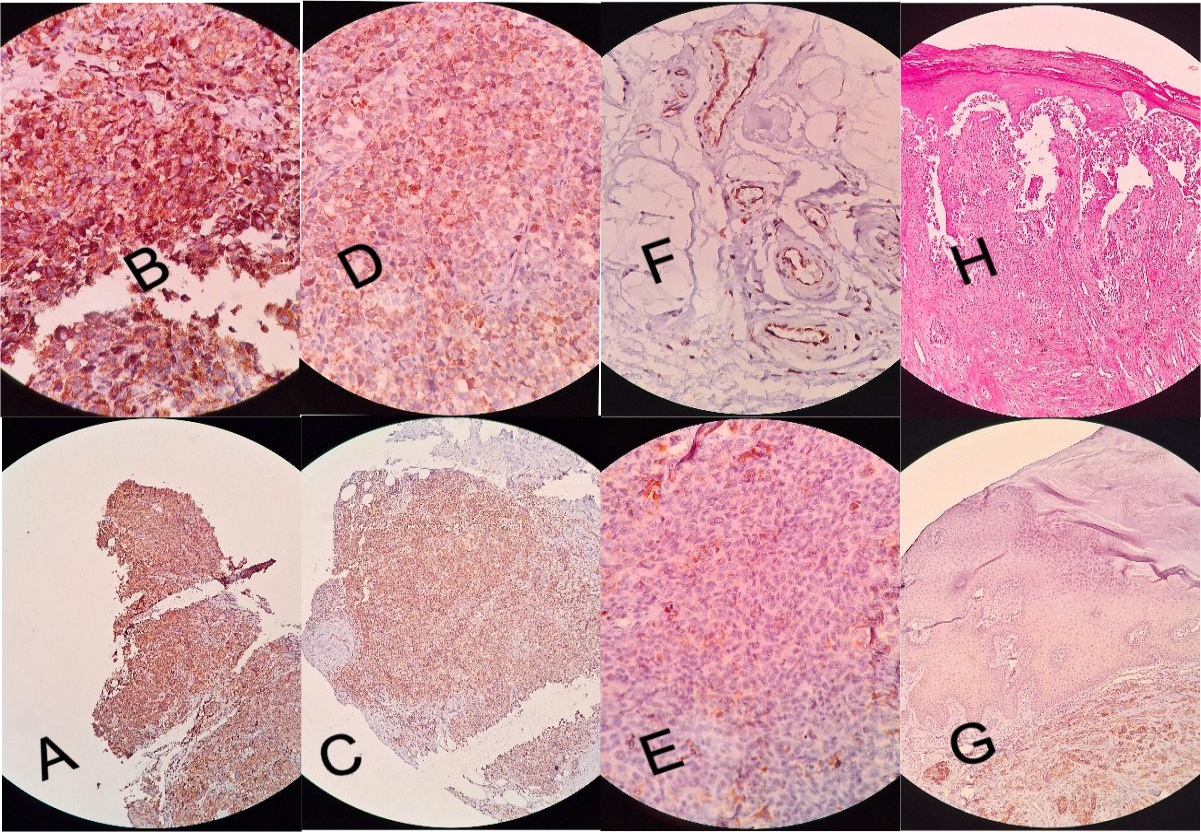
Immunohistochemical expression patterns of COX-2 in cutaneous melanoma tissues: high cytoplasmic expression as strong intensity of staining in (A) low power field (LPF) and (B) high power field; low cytoplasmic expression as weak intensity of staining in (C) low power field (LPF) and (D) high power field; (E) negative expression of COX-2, (F) cytoplasmic staining of endothelial cells as positive internal control, (G) epidermal cells as negative internal control, (H) H&E staining of melanoma tissues.

In addition, the results of ANOVA analysis showed that there was no significant relationship between the age of patients with the intensity (P = .284) and percentage of COX-2 staining (P = .307). In addition, the number of mitoses counted in each sample did not show any significant relationship with the intensity and percentage of COX-2 staining (P = .532 and P = .388, respectively). Figure 1 (A-D) shows weak and strong immunohistochemical expression of COX-2 in tumor cells of cutaneous melanoma.

**Table 2:** Shows the association between COX-2 expression and clinicopathological parameters of malignant melanoma (MM)

Patients and tumor characteristics	Intensity of staining N (%)			*P-value*	Percentage of staining N (%)			*P-value*
	Negative	Weak	Strong		<10%	10-50%	(>50%)	
Gender Male Female	3 (15.0) 2 (10.5)	6 (30.0) 4 (21.1)	11 (55.0) 13 (68.4)	0.690	3 (15.0) 2 (10.5)	4 (20.0) 5 (26.3)	13 (65.0) 12 (63.2)	0.850
Ulcer Yes No	2 (15.4) 2 (9.1)	2 (15.4) 5 (22.7)	9 (69.2) 15 (68.2)	0.777	2 (15.4) 2 (9.10)	1 (7.70) 6 (27.3)	10 (76.9) 14 (63.6)	0.357
Histopathology subtype Superficial spreading Lentigo maligna Desmoplastic Acral Nodular NOS In situ Metastatic	1 (16.7) 1 (20.0) 0 (0.00) 1 (8.3) 1 (12.5) 0 (0.00) 1 (33.3) 0 (0.00)	0 (0.00) 2 (40.0) 0 (0.00) 4 (33.3) 0 (0.00) 1 (33.3) 2 (66.7) 1(100)	5 (83.3) 2 (40.0) 1(100) 7 (58.3) 7 (87.5) 2 (66.7) 0 (0.00) 0 (0.00)	0.361	1 (16.7) 1 (20.0) 0 (0.00) 1 (8.3) 1 (12.5) 0 (0.00) 1 (33.3) 0 (0.00)	0 (0.00) 3 (60.0) 0 (0.00) 3 (25.0) 0 (0.00) 1 (33.3) 2 (66.7) 0 (0.00)	5 (83.3) 1 (20.0) 1(100) 8 (66.7) 7 (87.5) 2 (66.7) 0 (0.00) 1(100)	0.299
Tumor location Outer ear Face Scalp and neck trunk Upper limb Lower limb	0 (0.00) 0 (0.00) 1 (14.3) 2 (50.0) 2 (22.2) 0 (0.00)	0 (0.00) 3 (60.0) 1 (14.3) 0 (0.00) 1 (11.1) 5 (38.5)	1 (100) 2 (40.0) 5 (71.4) 2 (50.0) 6 (66.7) 8 (61.5)	0.185	0 (0.00) 0 (0.00) 1 (14.3) 2 (50.0) 2 (22.2) 0 (0.00)	0 (0.00) 4 (80.0) 1 (14.3) 0 (0.00) 1 (11.1) 3 (23.1)	1(100) 1 (20.0) 5 (71.4) 2 (50.0) 6 (66.7) 10 (76.9)	**0.046**
Clark level I II III IV V	1 (100) 1 (14.3) 2 (22.2) 1 (6.7) 0 (0.00)	0 (0.00) 1 (14.3) 2 (22.2) 4 (26.7) 0 (0.00)	0 (0.00) 5 (71.4) 5 (55.6) 10 (66.6) 2 (100)	0.446	0 (0.00) 1 (14.3) 2 (22.2) 1 (6.70) 0 (0.00)	0 (0.00) 1 (14.3) 2 (22.2) 3 (20.0) 0 (0.00)	1(100) 5 (71.4) 5 (55.6) 11 (73.3) 2 (100)	0.444
Vascular invasion Yes No	0 (0.00) 4 (12.1)	0 (0.00) 7 (21.2)	2 (100.0) 22 (66.7)	0.615	0 (0.00) 4 (12.1)	0 (0.00) 7 (21.2)	2 (100.0) 22 (66.7)	0.615
Breslow thickness ≤2 mm >2 mm	1 (5.6) 2 (14.3)	5 (27.8) 2 (14.3)	12 (66.7) 10 (71.4)	0.516	1 (5.6) 2 (14.3)	5 (27.8) 1 (7.10)	12 (66.7) 11 (78.6)	0.275
Intra-tumoral lymphocytes Negative Low High	1 (6.7) 3 (18.8) 0 (0.00)	2 (13.3) 4 (25.0) 1 (25.0)	12 (80.0) 9 (56.3) 3 (75.0)	0.600	1 (6.7) 3 (18.8) 0 (0.00)	4 (26.7) 3 (18.8) 0 (0.00)	10 (66.7) 10 (62.5) 4 (100)	0.506
T stage 1 2 3 4	1 (16.7) 0 (0.00) 1 (8.30) 1 (25.0)	1 (16.7) 2 (20.0) 4 (33.3) 0 (0.00)	4 (66.7) 8 (80.0) 7 (58.3) 3 (75.0)	0.620	1 (16.7) 0 (0.00) 1 (8.30) 1 (25.0)	0 (0.00) 3 (30.0) 3 (25.0) 0 (0.00)	5 (83.3) 7 (70.0) 8 (66.7) 3 (75.0)	0.506
Elastosis Negative Grade I Grade II	5 (14.7) 0 (0.00) 0 (0.00)	9 (26.5) 1 (25.0) 0 (0.00)	20 (58.8) 3 (75.0) 1 (100)	0.844	5 (14.7) 0 (0.00) 0 (0.00)	8 (23.5) 1 (25.0) 0 (0.00)	21 (61.8) 3 (75.0) 1 (100)	0.865

samples (Intensity and percentage of staining) (*P* *value*; Pearson’s χ2 test).

**Values in bold are statistically **significant.

## Discussion

Melanomas constitute a significant health care concern, and early diagnosis is important for managing treatment, decreasing treatment costs, and enhancing follow-up (38).

Considering the high incidence of melanoma worldwide, including in Iran, early diagnosis and the identification of novel therapeutic approaches to control and eliminate disease progression are essential. Inducible COX-2 has been attributed to numerous functions in the biology of several carcinomas, including increased cell proliferation (39), angiogenesis (40), and inhibition of immunosurveillance and apoptosis (41, 42).

In the present study, the expression level of COX-2, an effective enzyme in inflammatory and immunological responses, was investigated in tissue samples of human melanoma using IHC. Our findings showed that approximately 87% of MM showed COX-2 protein expression: 61.5% of cases with strong expression and 64% of cases with staining in more than 50% of tumor cells. Studies on COX-2 expression in other tumor types support this observation. Ghasemi et al. (38) observed COX-2 expression in all skin melanoma samples, with 65% of cases showing strong expression.

By immunohistochemical examination, Becker et al. (43) reported strong COX-2 expression in 95% of primary melanoma tumors. Denkert et al. (42) reported COX-2 expression in 93% of MM cases, with strong and moderate expression in 68% of cases.

Meanwhile, other studies have reported lower COX-2 expression in skin melanoma samples. One study showed COX-2 expression in 50% of acral melanoma samples (44). Iacono (45) observed COX-2 expression in 30% of cutaneous melanoma cases in patients older than 65 years. However, only patients in the early stages of the disease were included and evaluated (45). Minsini et al. (46) reported COX-2 expression in only 28% of skin melanoma cases.

Overall, factors such as differences in the statistical population, the use of different clones of COX-2 antibody for IHC staining, and differences in the interpretation and grading of COX-2 expression can affect the results of different studies. Despite the differences in COX-2 expression levels in melanoma tumor tissues, most studies showed higher expression of this marker in MM than in benign melanocytic lesions (38, 42, 47). Kuzbicki et al. (48) found that the average level of COX-2 expression in melanoma samples was significantly higher than that in benign nevi, and COX-2 expression in tumor tissues with lymph node metastasis was significantly higher than that in non-metastatic melanoma (48). These findings suggest a possible role for COX-2 overexpression in the development and progression of MM.

Therefore, considering the high expression of COX-2 in MM tumors, especially in advanced and metastatic stages, targeted therapy using selective COX-2 inhibitory antibodies or NSAIDs may have a potential role in treating melanoma patients, particularly those with malignant types. Melanoma cells often evade immune responses and develop resistance to cancer immunotherapy through increased expression of COX-2 and PDL-1 (49). Celecoxib may be effective in improving the response to chemotherapy in melanoma patients by negatively regulating COX-2 expression and inducing apoptosis in melanoma cells via oxygen free radicals (49).

In this study, the level of COX-2 expression in benign melanocytic lesions was not examined. However, its expression is much lower in melanoma in situ and Lentigo maligna, which have a lower malignant potential compared with more aggressive subtypes such as nodular, superficial spreading, and acral melanoma, which was not statistically significant. None of the three in situ melanoma tumors examined in our study showed strong COX-2 expression in more than 50% of tumor cells. In Lentigo maligna, strong COX-2 staining was observed in 40% of samples, and only 20% of cases showed COX-2 expression in more than 50% of tumor cells. More than 80% of cases with nodular and superficial spreading MM tumors showed strong expression in more than 50% of tumor cells. Nevertheless, all observed differences were not statistically significant, possibly due to the small sample size, especially in the melanoma in situ and lentigo maligna subtypes.

Consistent with other studies, there was no statistically significant correlation between COX-2 expression level and tumor histological subtype (43, 45). Lee et al. (50) also found no significant association between COX-2 expression and specific pathological subgroups of MM. In contrast, Kuzbicki et al. (48) reported significantly higher expression of COX-2 in nodular melanomas than in superficial spreading melanomas, contrary to our results. Denkert et al. (42) observed stronger COX-2 expression in the nodular melanoma subgroup, but the difference with other groups was not statistically significant. In our study, COX-2 expression was slightly higher in nodular melanomas than in other subtypes, possibly due to the Breslow thickness of the tumor in this subtype compared with other melanoma subtypes.

Overall, various studies have shown that COX-2 expression level may differ based on the type of lesion, such as reduced expression in benign moles, rare expression in dysplastic lesions, and increased expression in melanomas. Similarly, primary melanomas have a lower COX-2 expression level than metastatic melanomas (51).

Evaluation of the clinical and histopathological significance of COX-2 overexpression with factors related to melanoma prognosis did not show any significant statistical association, and only tumor location had clinical significance. Tumors in the facial area had lower COX-2 expression compared with tumors in other organs, which could explain the greater number of Lentigo maligna lesions in the facial area in our study, as well as the lower skin thickness in this area.

Based on our study's findings, there was no statistically significant relationship between the intensity and percentage of COX-2 expression and the depth of tumor invasion based on Clark’s levels and Breslow tumor thickness. However, there was an increasing trend in COX-2 expression level with increasing Clark tumor level, and tumors with Clark levels IV and V showed strong expression of this marker in more than 50% of tumor cells. Minami et al. (47) showed a non-significant trend in COX-2 expression intensity with increasing depth of tumor invasion. Iacono et al. (45) also did not indicate a statistically significant relationship between COX-2 expression level and invasion in terms of Clark's level and tumor thickness, similar to our results.

Becker et al. (72) found no statistically significant correlation between COX-2 expression and tumor Clark level, but its overexpression was related to increased tumor thickness. However, several studies have shown a significant correlation between COX-2 expression intensity, Clark level, and tumor invasion. Contrary to our results, one study showed a strong significant correlation between COX-2 expression level and Clark level and tumor thickness (48).

Another study reported a statistically significant correlation between COX-2 expression and tumor invasion in acral melanomas (44). A significant correlation was reported in skin melanoma between COX-2 overexpression and advanced Clark stages (52). According to this trend, the level of significance in our results may improve with a larger sample size.

Another histological factor affecting melanoma prognosis, which has been related to COX-2 expression level in some studies, is the number of mitoses. However, in our study, there was no statistically significant relationship between the number of mitoses and the severity of COX-2 expression in tumor cells, consistent with another study (45). However, two studies reported a significant positive correlation between the number of mitoses and increased COX-2 expression (46, 53). These differences could be due to variations in the characteristics and histological subtypes of the tumors across the studies.

Considering the importance of optical damage in the new WHO classification, the presence and severity of elastosis in tumor tissues was one of the histological variables investigated in our study. An increasing, but not statistically significant, trend in COX-2 expression intensity and percentage was observed with an increasing degree of elastosis. In the current study, elastosis was observed in only five tumors, limiting definitive conclusions in this area.

Moreover, several studies have reported dissimilar findings regarding clinical and histological variables such as age, sex, presence of wound, tumor stage, vascular invasion, and intra-tumor lymphocytes. Ulcerated melanomas showed COX-2 expression at a higher level than non-ulcerated melanomas (48). Kuzbicki et al. (48) also reported that COX-2 expression level is related to tumor stage. However, other studies, including ours, found no statistically significant correlation in this area (42, 45).

In this study, survival outcomes and recurrence in patients with MM were not investigated. Previously published studies have reported an association between higher COX-2 expression and decreased survival in patients with melanoma. Shorter disease-specific survival was associated with COX-2 overexpression in patients with MM (43, 46, 52). These findings suggest that COX-2 is an adverse prognostic marker in cutaneous melanoma, although our study did not show a significant relationship between the histopathological markers affecting MM prognosis.

The limitations of the present study include its retrospective nature and the average sample size, which make it impossible to evaluate the clinical and long-term outcomes of the patients. Additionally, the small sample size in some comparative subgroups limits the possibility of drawing accurate conclusions in some cases. Furthermore, the limited information available in the patients’ documents prevented the investigation of factors related to the patients’ prognosis, such as lymph node involvement and distant metastasis. We used the immunohistochemical method, which shows COX-2 expression level at the protein level. A detailed examination of the gene expression of this marker requires molecular studies such as real-time PCR and Western blotting.

## Conclusion

Our study found that COX-2 expression is strongly correlated with anatomic location in most MMs. There were no associations between gender, ulcerative lesions, melanoma subtypes, tumor thickness, vascular invasion, lymphocyte infiltration, mitoses, and elastosis. Therefore, the prognostic significance of COX-2 remains unclear. In patients with MM and COX-2 overexpression, prospective studies may explore clinical outcomes and evaluate the effects of COX-2 inhibitors as single or combination therapies.

## Abbreviations

*COXs*: Cyclooxygenases

*COX-2: *Cyclooxygenase-2

*MM: *Malignant Melanoma

*UV:* Ultraviolet

*PG:* Prostaglandins

*EGF:* Epidermal Growth Factor

*CSC:* Cancer Stem Cells

*FFPE*: Formalin-Fixed Paraffin-Embedded

*H&E*: Hematoxylin and Eosin

*WHO*: World Health Organization

*IHC*: Immunohistochemistry

## Data Availability

Data are available upon reasonable request from the corresponding author.
